# Late-onset dementia: a mosaic of prototypical pathologies modifiable by diet and lifestyle

**DOI:** 10.1038/npjamd.2015.3

**Published:** 2015-09-28

**Authors:** Mark P Mattson

**Affiliations:** 1 Laboratory of Neurosciences, National Institute on Aging Intramural Research Program, Baltimore, MD, USA; 2 Department of Neuroscience, Johns Hopkins University School of Medicine, Baltimore, MD, USA

## Abstract

Idiopathic late-onset dementia (ILOD) describes impairments of memory, reasoning and/or social abilities in the elderly that compromise their daily functioning. Dementia occurs in several major prototypical neurodegenerative disorders that are currently defined by neuropathological criteria, most notably Alzheimer’s disease (AD), Lewy body dementia (LBD), frontotemporal dementia (FTD) and hippocampal sclerosis of aging (HSA). However, people who die with ILOD commonly exhibit mixed pathologies that vary within and between brain regions. Indeed, many patients diagnosed with probable AD exhibit only modest amounts of disease-defining amyloid β-peptide plaques and p-Tau tangles, and may have features of FTD (TDP-43 inclusions), Parkinson’s disease (α-synuclein accumulation), HSA and vascular lesions. Here I argue that this ‘mosaic neuropathological landscape’ is the result of commonalities in aging-related processes that render neurons vulnerable to the entire spectrum of ILODs. In this view, all ILODs involve deficits in neuronal energy metabolism, neurotrophic signaling and adaptive cellular stress responses, and associated dysregulation of neuronal calcium handling and autophagy. Although this mosaic of neuropathologies and underlying mechanisms poses major hurdles for development of disease-specific therapeutic interventions, it also suggests that certain interventions would be beneficial for all ILODs. Indeed, emerging evidence suggests that the brain can be protected against ILOD by lifelong intermittent physiological challenges including exercise, energy restriction and intellectual endeavors; these interventions enhance cellular stress resistance and facilitate neuroplasticity. There is also therapeutic potential for interventions that bolster neuronal bioenergetics and/or activate one or more adaptive cellular stress response pathways in brain cells. A wider appreciation that all ILODs share age-related cellular and molecular alterations upstream of aggregated protein lesions, and that these upstream events can be mitigated, may lead to implementation of novel intervention strategies aimed at reversing the rising tide of ILODs.

## Histopathological landscapes of prototypical dementias

The brain regions that suffer the greatest amount of synapse loss and neuronal death in ILOD include the hippocampus, entorhinal cortex, medial temporal lobe, frontal cortex and inferior parietal cortex. However, the amount of neurodegeneration varies considerably between brain regions and among individuals, which may explain, in part, the inter-individual variability in the type and magnitude of deficits in different cognitive domains.^1–3^ Neurons that degenerate often exhibit accumulations of aggregated proteins that form fibrillar or more amorphous inclusions within their cell bodies and neurites, and/or form extracellular deposits of aggregated proteins. Together with an understanding of the genetic causes of rare cases of Alzheimer’s disease (AD), Parkinson’s disease (PD) and FTD, the specific proteins that accumulate within or outside of cells in the affected brain regions have been used to classify some ILODs as specific diseases. For example, a diagnosis of AD is ultimately established by semiquantitative analysis of neurofibrillary tangles (comprised of hyperphosphorylated Tau protein; p-Tau) and the density of large extracellular aggregates of amyloid β-peptide (Aβ) that form amyloid plaques. Mutations in the β-amyloid precursor protein (APP) and presenilin 1 that cause early-onset familial AD result in increased production of aggregation-prone neurotoxic forms of Aβ, neurofibrillary tangle formation and associated neuronal death.^4^ However, whereas in late-onset AD the amyloid and p-Tau pathologies occur predominantly in cerebral cortical regions, in familial AD subcortical structures such as the striatum are also often severely affected.^5^ In this section, I describe key histopathological criteria used to assign a specific/prototypical disease diagnosis to ILOD patients, and also briefly summarize genetic aberrancies that are known to cause familial early-onset dementias. As there exists an immense literature on these disorders, I reference mainly review articles in which key original research articles are cited.

### Alzheimer’s disease

**Figure 1:**
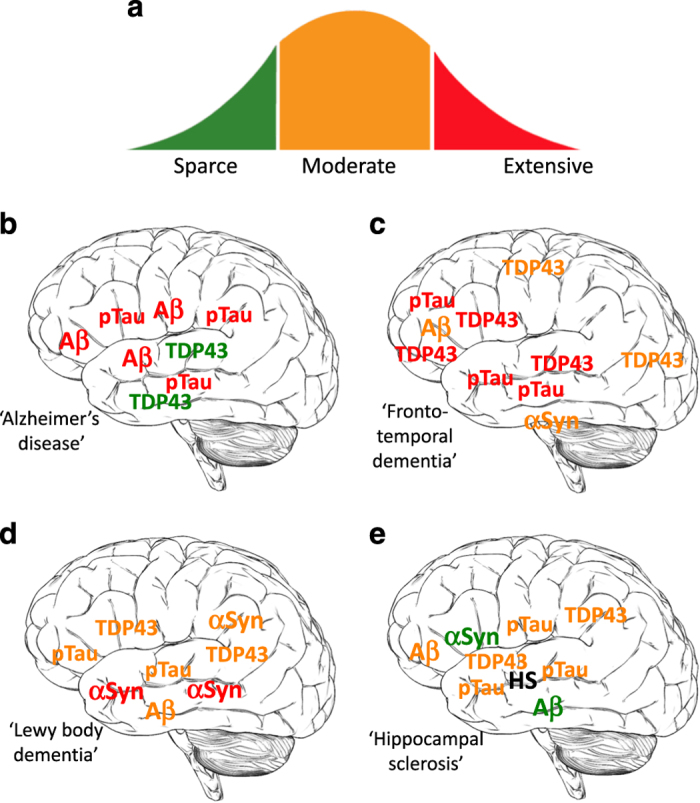
Each case of idiopathic late-onset dementia is a unique mosaic of prototypical neuropathological landscapes. (**a**) Among individuals with idiopathic late-onset dementia (ILOD) there are variable amounts of proteopathic protein aggregates from absent/sparse (green) to moderate (orange) to extensive (red): amyloid β-peptide (Aβ), hyperphosphorylated Tau (pTau), TDP-43 (TDP43) and α-synuclein (αSyn). (**b**–**e**) Examples of neuropathological landscapes of four different patients with ILOD. (**b**) This patient exhibits robust Alzheimer’s disease pathology in the temporal, frontal and inferior parietal cortices with extensive Aβ and pTau pathologies. (**c**) This case is dominated by TDP-43 and p-Tau pathologies in the frontal and temporal lobes, with lesser amounts of TDP-43 in sensory and motor regions of the cerebral cortex, and moderate amounts of α-synuclein in the brainstem. (**d**) This patient exhibits prominent Lewy body (α-synuclein) pathology and moderate amounts of Aβ and pTau pathologies. (**e**) In some cases of ILOD there are low to moderate amounts of each of the four pathogenic proteins in the temporal, frontal and inferior parietal cortices, and extensive hippocampal sclerosis (HS).

A diagnosis of AD requires that the patient has a clinical history of progressive memory impairment and exhibits two defining histopathological features of AD, the presence of abundant extracellular Aβ plaques that often exhibit dystrophic neurites (neuritic plaques), and intraneuronal fibrillar aggregates of p-Tau (neurofibrillary tangles) (Figure 1a,b). The severity of the Aβ plaque pathology is ranked from minimal (Thal stage 1) to severe (Thal stage 5), and the neurofibrillary tangle pathology is ranked on a 6-point scale from minimal (Braak stage I) to severe (Braak stage VI).^6^ However, many cognitively normal elderly subjects harbor as much amyloid pathology as AD patients,^7^ suggesting that their neurons are able to withstand the neurotoxic effects of Aβ.

Studies of families in which early-onset AD is inherited in an autosomal dominant manner have identified three genes that harbor genetic aberrancies, namely, presenilin 1, presenilin 2 and the β-APP.^4^ These mutations accelerate Aβ accumulation by altering enzymatic processing of APP, and evidence suggests that the mutations also result in dysregulation of neuronal calcium handling, which may render neurons vulnerable to age-related oxidative stress and metabolic deficits.^8^ Subjects with familial AD may live for 40–50 years without evidence of cognitive impairment, indicating that, even with a disease-causing mutation, the underlying neuropathological alterations evolve over an extended time period. However, familial AD accounts for a very small percentage of all cases of AD and it is as yet unclear whether altered proteolytic processing of APP is a pivotal early event or a later ‘disease-accelerating’ event in the common cases of sporadic AD. Beyond disease-causing mutations, there is one established genetic risk factor (ApoE 4) and several candidate genetic risk factors for AD.^4^

### Frontotemporal dementia

Some inherited cases of FTD are caused by mutations in the gene encoding Tau. While progressive dementia occurs in FTD, many patients first present with psychiatric symptoms such as apathy, impulsive behavior and aggressiveness. Motor dysfunction similar to PD also occurs in many FTD patients. Cognitive symptoms include attention deficits and executive dysfunction. There is considerable inter-individual variability in the clinical presentation of FTD patients, even within the same family.^9^ The histopathological features of FTD are dominated by intraneuronal accumulation of p-Tau amorphous aggregates and filaments. There is little or no Aβ pathology in FTD (Figure 1c). The p-Tau pathology is usually confined to the cerebral cortex gray matter and white matter. Atrophy of the frontal and temporal lobes is severe. Mutations in the gene encoding transactive response DNA-binding protein 43 (TDP-43) cause some cases of FTD,^10^ and cytoplasmic inclusions containing phosphorylated and ubiquitinated forms of TDP-43 are prominent in FTD.^10^ Recently, it was found that hexanucleotide repeat expansions in C9ORF72 (a non-coding region on chromosome 9) are responsible for many cases of inherited and sporadic FTD and amyotrophic lateral sclerosis.^11^ TDP-43 pathology is a prominent feature in patients with C9ORF72 hexanucleotide repeat expansions. In addition, recent findings suggest that the C9ORF72 hexanucleotide repeat DNA is transcribed and the mRNA is translated into dipeptide repeat proteins that aggregate and can be neurotoxic.^12^

### Hippocampal sclerosis of aging

Progressive atrophy of the hippocampus is a common feature of AD that is associated with cognitive decline. However, many subjects with extensive hippocampal atrophy, and who are clinically indistinguishable from AD patients, exhibit only minimal Aβ plaque and p-Tau pathologies. Instead these patients suffer massive neuronal loss and gliosis in the CA1 region of the hippocampus and the adjacent subiculum, the defining pathology of HSA.^13^ HSA is most common in patients over the age of 80, affecting >20% of those diagnosed with probable AD. While Aβ plaque and p-Tau pathologies are not prominent in HSA, TDP-43 pathology is common, with TDP-43 cytoplasmic inclusions occurring in neurons and astrocytes of the hippocampus and associated neocortical regions in the frontal and temporal lobes, and the amygdala.

The genetics of HSA is largely unexplored. Families with Mendelian inheritance of HSA have not been reported. However, recent studies have associated single-nucleotide polymorphisms in genes that encode the mitochondrial potassium channel protein ABCC9, the growth factor-like glycoprotein progranulin and the lysosome/autophagy-associated protein TMEM106B with increased risk of HSA.^14–16^ These findings suggest that alterations in the regulation of neuronal excitability, neurotrophic support and lysosome function contribute to the pathogenesis of HSA, consistent with the notion that all ILODs involve impaired neuronal energy metabolism, neurotrophic signaling, calcium handling and autophagy.

### LBD and PD dementia

The symptoms of patients with LBD include cognitive impairment, hallucinations, depression, intermittent confusion and PD-like motor signs (bradykinesia, rigidity and myoclonus). Aggregates of α-synuclein in the cytoplasm of neurons throughout the cerebral cortex and subcortical structures is the characteristic histopathological feature of LBD17 (Figure 1d). In some neurons the α-synuclein aggregates completely fill the cytoplasm of the cell body (so-called ‘Lewy bodies’), whereas in other neurons smaller granular aggregates are evident. Notably, α-synuclein aggregates are also evident in axon terminals where they may compromise synaptic function. Mutations in the genes encoding α-synuclein and glucocerebrosidase can cause rare cases of LBD.^18^

Up to 75% of PD patients will develop clinical dementia, which typically occurs after the onset of autonomic and motor symptoms. Compared with age-matched control subjects, and dementia-free PD patients, PD patients with dementia exhibit cortical atrophy, white matter abnormalities and elevated amounts of Aβ pathology.^19^ Major advances in understanding the pathogenesis of PD have come from geneticists and neuroscientists who have identified and characterized several prominent mutations that cause PD.^20^ Autosomal dominant PD has been linked to mutations in α-synuclein, leucine-rich repeat kinase 2 and vacuolar protein sorting-associated protein 35 (VPS35). Autosomal recessive causes of PD include mutations in the genes encoding Parkin, phosphatase and tensin homolog (PTEN)-induced kinase 1 (PINK1), DJ1 and ATP13A2. Remarkably, accrued knowledge of the apparent normal functions of these proteins and the impact of the PD causal mutations on neurons provides convincing evidence that neuronal degeneration in PD involves impaired mitochondrial bioenergetics and a compromised ability of neurons to remove α-synuclein via proteasome- and lysosome-mediated mechanisms. The centrality of mitochondrial dysfunction in PD is underscored by the fact that mitochondrial toxins can cause selective degeneration of dopaminergic neurons and associated motor symptoms that are very similar to inherited and idiopathic PD.^21^

## Mosaic neuropathological landscapes are common in ILOD

The heterogeneity of the neuropathological landscape of ILOD is now widely appreciated and described in the literature.^2,22–25^ The presence of specific aggregated and posttranslationally modified (hyperphosphorylated and poly-ubiquitinated) proteins has been used to classify the cellular pathology of ILOD with the intention of labeling individual patients with a disease diagnosis. Early molecular genetic studies of AD, PD and FTD provided justification for the latter approach because APP mutations can cause dementia, α-synuclein mutations can cause PD and Tau mutations can cause FTD; the affected individuals exhibit predominantly Aβ, α-synuclein and Tau pathologies, respectively. However, many or perhaps most cases of ILOD are not readily placed within an AD, PD or FTD ‘disease bin’. Instead, there is a mosaic of histopathological phenotypes among ILOD patients (Figure 1). For example, in some cases, TDP-43 inclusions and HSA may be robust, with Aβ and p-Tau pathologies minimal. Other ILOD patients with a similar cognitive deficit profile may have abundant neuritic plaques and neurofibrillary tangles in the entorhinal cortex, hippocampus and inferior parietal cortex, and so are given a diagnosis of AD. Patients with LBD often exhibit hippocampal sclerosis and TDP-43 inclusions in hippocampal neurons.^26^ In one study of 342 subjects diagnosed with AD based on Aβ and p-Tau pathology, 195 of the subjects exhibited TDP-43 pathology.^23^ In the latter study, TDP-43 pathology was strongly correlated with cognitive impairment and medial temporal lobe atrophy.

The blurring of the lines between what had once been considered discrete diseases is further emphasized by the existence of inherited cases of ILOD caused by mutations in the same gene, but exhibiting distinct neuropathological landscapes. For example, whereas most APP mutations that cause familial AD exhibit robust Aβ plaques and p-Tau tangles in hippocampus, entorhinal cortex, and frontal and temporal lobes, members of a family with an APP V717I mutation exhibited extensive α-synuclein Lewy body pathology in some regions of neocortex and the substantia nigra.^27^ Interestingly, affected members of the latter family also exhibited robust Aβ and p-Tau pathology in the primary visual cortex, a brain region not usually affected in AD. Some presenilin 1 mutations also result in a mixed pathology with features of both AD and PD, often with distributions not typical of late-onset AD and ILODs. For example, affected members of a family with the presenilin 1 S170F mutation developed dementia in their third decade of life and, in addition to classic AD pathology, they exhibited Lewy bodies in the brainstem, limbic structures and neocortex.^28^ The mixed neuropathologies among subjects with different mutations in the same gene strongly suggest that other genetic factors and environmental factors impact the disease process.

In ILOD, advancing age is the major risk factor and provides a progressively unfavorable environment within the brain, and evidence from studies of animal models and human subjects suggests that the development of such a pro-neurodegenerative cellular environment can be accelerated or retarded by genetic and environmental factors.^29,30^ While the majority of dementia patients exhibit pathology commensurate with the severity of their cognitive impairment, there is often discordance between the type and magnitude of the histopathological abnormalities and cognitive function. At one extreme are individuals with abundant Aβ pathology who are cognitively normal, and at the other extreme are those with extensive neuronal degeneration but relatively modest accumulation of proteopathic proteins.^31,32^ And so for each type of proteopathic alteration (Aβ plaques, p-Tau, α-synuclein aggregates, TDP-43 inclusions) any particular ILOD patient can be positioned somewhere within the distribution range of all ILOD patients (Figure 1). Assuming each of the different aggregation-prone proteins contributes to the dysfunction and degeneration of neurons in ILODs, then it follows that there are additional factors that determine whether or not neurons succumb to the cytotoxic action of the proteopathic protein(s). It should also be noted that a proteopathic protein may not be a critical factor in the mechanism of neuronal degeneration in some cases of ILOD; for example, the pathological landscape of HSA can be largely devoid of pathogenic protein aggregates. The remainder of this article considers the age-related cellular and molecular mechanisms that may render neurons vulnerable to ILOD, and how those mechanisms can be modified by environmental factors, with a focus on diet, exercise and intellectual challenges throughout the lifespan.

## Neuronal vulnerability in ILOD can occur upstream and downstream of proteopathic proteins

Histopathological studies of the brains of cognitively normal octogenarians, nonagenarians and centenarians have shown that essentially all very old subjects exhibit one or more neuropathological features, with many having levels of Aβ plaques and p-Tau sufficient for a diagnosis of AD.^31,32^ On the other hand, some subjects may have only modest amounts of Aβ plaques and p-Tau pathology and yet exhibit robust neuronal loss and cognitive impairment. These dissociations between levels of ‘pathogenic proteins’ and cognitive deficits strongly suggest that there are unknown factors that determine whether the neurons of any particular individual are resistant to or vulnerable to the accumulation of proteopathic proteins and/or are resistant to or vulnerable to the toxicity of the proteopathic proteins.

Considerable evidence suggests that synapse loss is a stronger predictor of cognitive impairment in ILOD than is any particular aggregated protein pathology.^33–36^ Knowledge of the qualitative and quantitative aspects of synapse physiology provides a framework for understanding why excitatory synapses may be the ‘Achilles heel’ of the neuronal networks that succumb in ILOD (and neurodegenerative disorders, in general). This topic has been reviewed in more detail elsewhere.^37–40^ Suffice it to say that excitatory (glutamatergic) synapses experience robust repetitive bouts of ionic, metabolic and oxidative stress during their normal activity throughout the life course. Synapse activation involves depolarization of the presynaptic terminal membrane, resulting in the opening of voltage-gated Na^+^ and Ca^2+^ channels, and Ca^2+^ influx, which triggers glutamate release from the presynaptic terminal. Glutamate activates postsynaptic ionotropic AMPA and N-methyl-D-aspartate (NMDA) receptors, resulting in Ca^2+^ influx and activation of kinases and transcription factors that regulate various acute and long-term adaptive responses of the neuron. Excessive sustained activation of glutamate receptors can cause degeneration of the synapse and neuronal death by activating proteases, impairing mitochondrial function and promoting oxidative stress.

**Figure 2:**
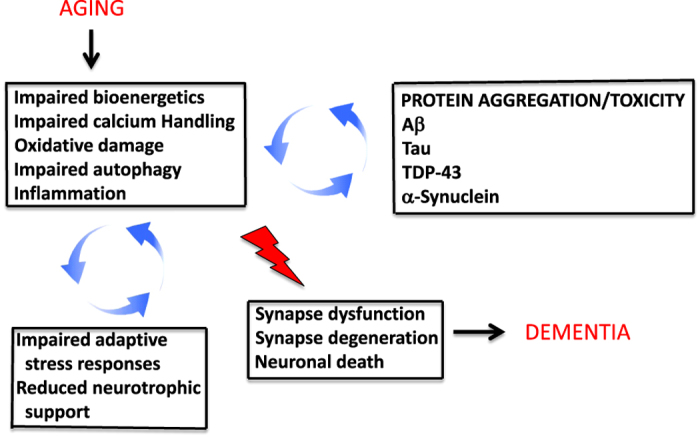
Generic age-related cellular stress and specific proteopathic abnormalities exert reciprocal cross-amplifying detrimental effects on synaptic plasticity and neuronal viability. During aging, neurons experience reduced energy availability (e.g., mitochondrial dysfunction and reduced glucose transport), increased levels of oxidative stress, perturbed cellular calcium homeostasis, impaired autophagy, and inflammation. The latter adverse changes are exacerbated by a reduced ability of neurons to respond adaptively to stress. The aggregation and associated neurotoxic activities of proteopathic proteins (Aβ, Tau, TDP-43 and α-synuclein) are promoted by metabolic, oxidative and calcium-related stress and impaired autophagy/protein degradation. Thus, cross-amplifying neurodegenerative processes result in synapse dysfunction, degeneration and neuronal death, resulting in dementia.

Early studies provided evidence that during the process of aggregation on the membrane of neurons and synapses Aβ causes lipid peroxidation, which impairs the function of ion-motive ATPases, and glucose and glutamate transporters, destabilizes Ca^2+^ homeostasis, and renders the neurons vulnerable to excitotoxicity.^41–44^ Importantly, several adverse conditions that occur in the brain during normal aging and that are exacerbated in ILOD may increase the vulnerability of neurons to the toxic actions of Aβ, p-Tau, α-synuclein and TDP-43 (Figure 2). These conditions include impaired bioenergetics/mitochondrial function, oxidative stress, inflammation, and impaired proteasome- and autophagy-mediated removal of damaged proteins and organelles.^45–50^ In addition, reductions in neurotrophic factor support as the result of decreased expression of the trophic factors and/or impaired signaling downstream of the neurotrophic factor receptors occurs during brain aging and more so in ILOD.^51^ For example, reduced expression of brain-derived neurotrophic factor (BDNF) and impaired insulin/insulin-like growth factor signaling occurs in the hippocampus in aging and AD.^51,52^ Both BDNF and insulin-like growth factor 1 can protect neurons in experimental models of AD.^53,54^

Data suggest that aging and stress-related cellular energy deficits, excessive activation of glutamate receptors and oxidative stress contribute to the accumulation of p-Tau and neurofibrillary degeneration.^55,56^ FTD-causing Tau mutations may promote neuronal degeneration by perturbing cellular Ca^2+^ regulation^57^ and impairing autophagy.^58^ Recent studies suggest that pathogenic forms of TDP-43 render neurons vulnerable to excitotoxicity^59^ and mitochondrial dysfunction^60,61^ and that stimulation of autophagy can protect neurons against TDP-43 toxicity.^62^ Finally, α-synuclein pathology may result from and exacerbate neuronal oxidative stress, mitochondrial dysfunction and impaired proteasome function and autophagy.^63,64^ It is likely that the latter alterations first compromise synapse function, which, in turn, leads to neuronal degeneration. Indeed, it was shown that α-synuclein pathology can be lessened, and synaptic dysfunction and memory impairment can be reversed, by inhibiting α-synuclein expression in an inducible α-synuclein transgenic mouse model.^65^

Altogether, the available data from studies of human subjects and experimental models suggest that oxidative stress, bioenergetic deficits, cellular Ca^2+^ dysregulation, impaired autophagy and inflammatory glial reactions (1) occur in the brain during normal aging; (2) initiate and accelerate the accumulation of proteopathic proteins (Aβ, p-Tau, α-synuclein and TDP-43) in ILOD; and (3) mediate the synaptotoxic and cell death-promoting effects of each of the different proteopathic proteins. This knowledge suggests that interventions that bolster neuronal bioenergetics, autophagy and defenses against oxidative and excitotoxic stress may forestall development of most, if not all, cases of ILOD.

## Compromised adaptive cellular stress responses and ILOD

The ability of neurons in the brain to cope with stress (bioenergetic, oxidative, ionic, proteotoxic) is diminished during aging.^66–70^ Although thousands of studies have documented age-related decrements in molecular mechanisms that promote neuronal plasticity and survival, most can be placed within one of a relatively few general categories of stress response pathways that include neurotrophic factor signaling; defense against oxidative stress; mitochondrial function; calcium homeostasis; protein quality control; molecular waste disposal; and DNA repair.

### Neurotrophic factors

Studies of postmortem brain tissue samples, and of animal and cell culture models, suggest that a decline in neurotrophic support contributes to the dysfunction and degeneration of neurons in ILODs. Among the neurotrophic factors that may be compromised in ILODs, BDNF is of particular interest because of its well-established fundamental roles in synaptic plasticity, learning and memory, and hippocampal neurogenesis.^71^ In human subjects levels of BDNF in the cerebrospinal fluid decrease during aging and are further reduced in subjects with poorer cognitive performance.^72^ Age-related reductions of BDNF expression in the hippocampus occur during normal aging in rodents, and further decrements in hippocampal BDNF levels have been reported to occur in animal models of AD.^73,74^ Moreover, when levels of BDNF are increased in the entorhinal cortex using a gene therapy approach, cognitive deficits can be reversed in rodent and non-human primate models of AD.^75^ BDNF deficiency is also implicated in the pathogenesis of PD,^71^ while roles for BDNF in HSA and LBD have yet to be investigated. BDNF may protect neurons against metabolic and oxidative stress by stimulating mitochondrial biogenesis,^76^ and by upregulating antioxidant defenses.^77^

### Antioxidant defenses

Superoxide dismutase 2 (SOD2) and heme oxygenase 1 are two antioxidant enzymes that decline in brain cells during aging.^77,78^ SOD2 protects neurons against decrements in energy availability and Aβ toxicity, and experimental reduction of SOD2 levels accelerates the onset of cognitive deficits in APP mutant transgenic AD mice.^79^ Moreover, that SOD2 deficiency can trigger Tau hyperphosphorylation^80^ suggests a role for elevated mitochondrial oxidative stress in AD, FTD and other ‘Tauopathies’. Healthy neurons respond to the oxidative stress triggered by excitatory synaptic activity by activating the transcription factors NF-κB and Nrf2, which, in turn induces the expression of SOD2 and heme oxygenase 1, respectively.^81,82^ Another defense against oxidative stress that is adversely impacted in aging and AD is the plasma membrane redox system, which includes the enzymes reduced form of nicotinamide adenine dinucleotide (NADH)-quinone oxidoreductase 1 (NQO1), NADH-ferrocyanide reductase, NADH-coenzyme Q10 reductase and NADH-cytochrome *c* reductase.^83,84^

### Cellular bioenergetics

Mitochondrial function generally declines during normal brain aging and to a greater extent in ILOD.^85–87^ Data suggest that electron transport chain proteins and proteins involved in the citric acid cycle are compromised in ILOD as a result of oxidative damage to the proteins and by damage to the mitochondrial DNA that encodes some of the electron transport chain proteins.^88^ Studies of patient brain tissue samples and of experimental models suggest that the alpha-ketoglutarate dehydrogenase complex of the citric acid cycle is adversely affected early in AD.^89^ In PD, complex I of the electron transport chain appears particularly prone to dysfunction.^86^ Emerging findings, elaborated upon below, suggest that interventions that enhance mitochondrial bioenergetics can counteract the neurodegenerative process in multiple ILODs including AD, PD dementia and FTD. For example, bolstering cellular bioenergetics by administration of nicotinamide^90^ or a ketone ester^91^ ameliorates learning and memory deficits in mouse models of AD. Moreover, a drug that opens mitochondrial potassium channels (diazoxide) can ameliorate cognitive defenses and lessen Aβ and p-Tau pathologies in a mouse model of AD.^92^ The latter finding is particularly interesting in light of recent evidence that polymorphisms in the gene encoding a subunit of the K^+^ channels activated by diazoxide may affect the risk of HSA.^15,16^

### Neuronal calcium handling

The ability of neurons to efficiently control the disposition of Ca^2+^ among subcellular compartments is compromised during aging and may result in Ca^2+^ overload and excitotoxic degeneration of synapses.^93,94^ It is well known that neurons are susceptible to excitotoxic damage when their bioenergetics is compromised and when they are subjected to increased levels of oxidative stress or reduced levels of neurotrophic support. One of the approved treatments for AD, memantine, acts by reducing Ca^2+^ influx through the NMDA subtype of glutamate receptor.^95^ Importantly, mutations in genes that cause early-onset AD (presenilin 1 and APP) and PD (α-synuclein and Parkin), and that may increase the risk of AD (ApoE 4) and HSA (ABCC9), impair the ability of neurons to properly regulate Ca^2+^ and may thereby render the neurons vulnerable to Ca^2+^ overload-mediated cell death.^8,9,96,97^ A better understanding of the pivotal molecular alterations that render neurons vulnerable to Ca^2+^ overload may suggest novel approaches for therapeutic interventions aimed at restoring cellular Ca^2+^ homeostasis.

### Molecular garbage disposal

A robust alteration that occurs in neurons during aging that likely contributes to the accumulation of proteopathic proteins in ILOD is impaired autophagy and proteasome function.^48,98^ Intracellular accumulation of Aβ, α-synuclein and Tau may normally be prevented be targeting these proteins to the proteasome and/or lysosomes. Impaired ubiquitin-mediated proteasomal degradation of α-synuclein may result from age-related oxidative damage to proteasome proteins^99^ or excessive production of α-synuclein.^100^ Impaired lysosome function is believed to contribute to the accumulation of dysfunctional mitochondria, which are normally degraded by ‘mitophagy’.^101^ Interventions that enhance proteasome function and autophagy have been reported to counteract neuronal dysfunction and degeneration in experimental models of ILODs. For example, methylene blue enhances proteasomal degradation and improves cognitive function in a mouse model of AD,^102^ and methylene blue also enhances autophagy and thereby suppresses Tau pathology in models relevant to FTD.^58^ Dietary energy restriction (DER), which is known to stimulate autophagy, is neuroprotective in animal models of AD and PD (see ref.^30^ for review). Thus, enhancement of protein quality control and removal of damaged proteins can counteract adverse conditions relevant to ILODs.

### DNA damage

DNA damage in brain cells is caused predominately by free radicals and is increased during normal aging, and to a greater extent in AD and presumably other ILODs. This accumulation of DNA damage results, in part, from impaired DNA repair, particularly base-excision repair.^88^ During aging there occurs an accumulation of damage to DNA in the nuclear genome and in mitochondria. A study of human subjects provided evidence that the regulatory elements of certain genes that encode proteins involved in synaptic plasticity and adaptive stress responses exhibit a predilection for DNA damage, resulting in a corresponding reduction in levels of expression of those genes.^103^ Studies of genetically modified mice have shown that deficiencies in several DNA repair enzymes increase the vulnerability of neurons to metabolic stress; examples include the base excision repair enzymes endonuclease VIII-like 1 and 7,8-dihydro-8-oxoguanine DNA glycosylase.^104,105^ In addition, expression of the enzyme DNA polymerase β decreases in brain cells during normal aging, and experimental reduction of DNA polymerase β expression triggers neuronal death and cognitive deficits in a mouse model of AD with Aβ and p-Tau pathology.^106^ It was recently reported that neuronal DNA repair can be enhanced by activation of glutamate receptors and BDNF,^107,108^ suggesting that age-related decrements in synaptic plasticity and neurotrophic support may contribute to increased neuronal DNA damage in ILOD.

## How might intermittent challenges bolster neuronal resistance to ILOD?

**Figure 3:**
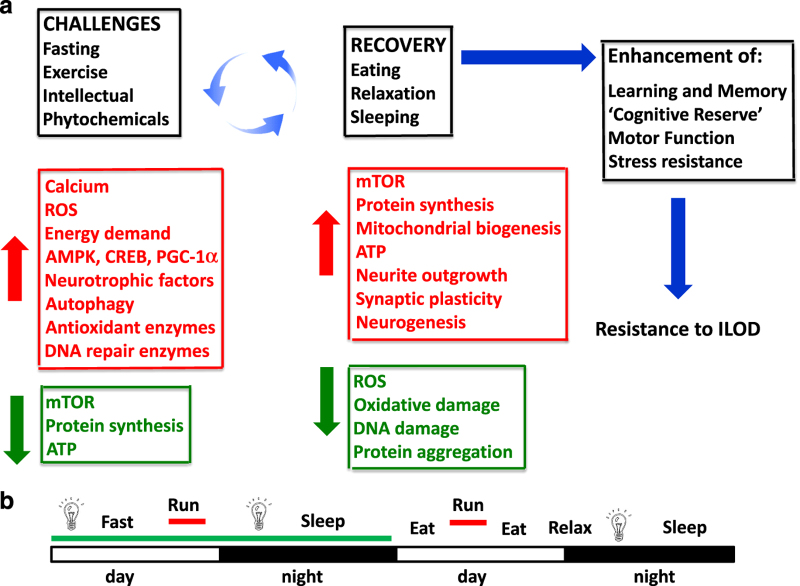
Intermittent bioenergetic challenges forestall ILOD by stimulating adaptive stress response pathways. (**a**) As with other species, humans evolved in environments where there was competition for food, mates and other resources. Accordingly, selection favored individuals whose brains functioned best when they were hungry, physically active and under stress. In response to the challenges (exercise, dietary energy restriction/fasting, intellectual challenges and consumption of noxious phytochemicals) neurons experience mild bioenergetic and oxidative stress. The neurons respond adaptively by activating signaling pathways that improve their ability to cope with more severe stress and resist disease. These neuroprotective pathways are triggered by calcium, reactive oxygen species (ROS) and increased energy demand, and involve kinases such as AMP-activated kinase (AMPK), and transcription factors such as cyclic AMP response element binding protein (CREB). The latter pathways increase autophagy, and induce the expression of genes encoding neurotrophic factors, antioxidant enzymes and DNA repair enzymes. During the challenges there is a reduction of mTOR (mammalian target of rapamycin) activity and protein synthesis. Once the challenge is over (e.g., food has been acquired) there is a recovery period that involves eating, relaxing and sleeping. During the recovery period mTOR activity, protein synthesis and mitochondrial biogenesis increase, and the growth of axons and dendrites, formation of new synapses and neurogenesis (the production of new neurons from stem cells) occur. Because of the adaptive stress responses induced during the challenge period levels of oxidative stress, DNA damage and protein aggregation are reduced. This model predicts that individuals who regularly engage in cycles of challenges and recovery periods during their adult life will exhibit optimal brain function and will be relatively resistant to the development of ILOD. (**b**) An example of a lifestyle that includes intermittent challenges as a means of optimizing brain health. In this case the person fasts (water or non-caloric beverages only) on the first day, while engaging in intellectual challenges (light bulb) and physical exercise (running). On the next day the subject eats several meals, runs, relaxes and engages in critical thinking.

In this section I summarize evidence that suggests it may be possible to forestall most cases of ILOD by enhancing the ability of neurons to mitigate the stressors involved in the neurodegenerative process, including metabolic, oxidative, ionic, proteotoxic and inflammatory stress. This can be accomplished by regular ‘challenge—recovery cycles’ in which neurons experience a mild stress (excitatory, metabolic and oxidative) during the challenge, followed by a rest/recovery period (Figure 3). For the following reasons, I focus on exercise, DER and regular engagement in intellectual challenges: (1) the evidence from animal studies that the latter three intermittent challenges enhance neuroplasticity and the resistance of neurons to injury and disease is compelling and conclusive; (2) the evidence from human studies is strong; (3) adaptive responses of the nervous system to exercise, energy restriction and intellectual challenges have been tested and refined during millions of years of evolution; (4) individuals concerned about their brain health can choose to incorporate these three challenges into their daily and weekly routines without concern for the kinds of adverse side effects common with drugs. For more thorough coverage of this topic, I refer the reader to recent reviews on exercise, energy restriction and intellectual challenges in brain health and disease resistance.^30,109–111^

Studies of human subjects have shown that regular physical exercise, particularly aerobic running, improves brain health as indicated by improved mood and enhanced cognitive function.^112,113^ Even in elderly subjects, exercise interventions can enhance cognitive performance and may preserve or increase gray and white matter volumes in some brain regions.^114,115^ Importantly, with regard to the main topic of the present article is evidence from human epidemiological data and studies of animal models suggesting that regular exercise can forestall ILOD and protein aggregation-related pathologies including Aβ, p-Tau and α-synuclein.^116–118^ Similarly, DER has been reported to ameliorate cognitive deficits and lessen accumulation and/or neurotoxicity of Aβ in animal models of AD.^119–121^ Environmental enrichment can also lessen Aβ and Tau pathologies and improve cognitive function in transgenic mouse models of ILOD.^122,123^ With regard to human studies there is evidence that individuals who avoid weight gain and central adiposity in midlife,^124,125^ and those who regularly engage in intellectually challenging endeavors,^126^ are at reduced risk for ILOD. The question then becomes 'what are the molecular and cellular mechanisms by which DER, exercise and cognitively stimulating environments can counteract age-related cellular stress and disease processes involved in ILOD?'

DER can robustly increase both the average and maximum lifespan in a range of mammalian species^127,128^ and can protect neurons against dysfunction and degeneration in animal models of AD, PD, Huntington’s disease, stroke and epileptic seizures.^30,129–131^ DER can also counteract the major age- and disease-related adverse conditions that may trigger and exacerbate ILOD, including oxidative stress,^132–134^ bioenergetic deficits,^135,136^ excitotoxicity,^129,137^ inflammation^30,138^ and proteotoxic pathologies.^130,131^ Compared to DER, voluntary running wheel exercise has more modest effects on lifespan,^139,140^ and on markers of brain oxidative stress and inflammation.^141–143^ Similar to exercise, environmental enrichment has been reported to reduce brain oxidative stress and inflammation in animal models of ILOD.^144,145^

Neurotrophic factors produced in an activity-dependent and cellular energy status-responsive manner mediate adaptive neuroplastic responses to exercise, fasting and cognitive enrichment. The cellular responses to these challenges include long-term potentiation of synaptic transmission, dendritic spine formation and hippocampal neurogenesis.^30^ Exercise and cognitive challenges induce BDNF expression in the hippocampus and other brain regions.^146–148^ The evidence that BDNF plays critical roles in multiple beneficial effects of exercise and enriched environments on hippocampal plasticity in mice and rats is extensive.^146–149^ Whether BDNF is critical for protection against ILOD by exercise, DER and intellectual challenges remains to be established. However, a recent study provided evidence that a socially enriched environment can rescue memory deficits in a mouse model of AD by a BDNF-dependent mechanism.^150^ Some studies have reported that exercise can increase BDNF levels in the serum or plasma of human subjects,^150^ although the source of the circulating BDNF is unknown, and it is unclear whether there is a direct relationship between brain and blood BDNF levels. In addition to BDNF, insulin-like growth factor 1 mediates adaptive responses of the brain to exercise. Circulating insulin-like growth factor 1 can enter the brain and affect gene expression in ways that stimulate neurogenesis and angiogenesis, and enhance synaptic plasticity and cognitive function.^151–153^

Much as occurs in skeletal muscle cells in response to vigorous exercise, recent findings suggest that bioenergetic challenges increase the ability of neurons to generate ATP by stimulating mitochondrial biogenesis (the growth and division of mitochondria). During exercise, Ca^2+^ influx and reactive oxygen species activate adenosine monophosphate-activated kinase (AMPK) and the transcription factor peroxisome proliferator-activated receptor-gamma coactivator 1α (PGC-1α) in muscle cells.^154^ PGC-1α then induces the expression of multiple genes encoding proteins required for mitochondrial biogenesis. In one study, treadmill training resulted in increased levels of PGC-1α and mitochondrial DNA electron transport chain proteins in multiple brain regions of mice, suggesting an increase in mitochondrial biogenesis.^155^ Combining intermittent exercise with DER enhances BDNF production and increases dendritic spine density in normal and diabetic mice,^142^ although it is unclear if DER enhances the effects of exercise on neuronal mitochondrial biogenesis. However, recent findings suggest that BDNF may mediate mitochondrial biogenesis in response to exercise, DER and cognitive challenges. As evidence, BDNF induces PGC-1α expression and mitochondrial biogenesis in hippocampal neurons.^76^ Moreover, the ability of BDNF to promote synapse formation requires PGC-1α expression,^76^ suggesting the possibility that mitochondrial biogenesis plays a role in the stimulation of synapse formation by exercise and DER, and the protection of synapses against degeneration in ILOD.

While it is evident that regular engagement in cognitive challenges can promote maintenance of cognitive abilities during aging, an understanding of why intermittent exercise and DER also enhance cognitive abilities and may forestall ILOD is clarified by evolutionary considerations. Our human ancestors, and the species that preceded them, were regularly challenged with the necessity of acquiring sufficient food resources for survival and to support reproduction. Presumably, individuals whose cognitive abilities were best when they were hungry, physically fit, and actively exploring and encoding mental maps of their environment would have a survival advantage. Nervous systems that responded adaptively to the challenge of competing for limited amounts of food were those that were selected for. Arguably, many of the higher cognitive abilities that humans now possess evolved for the purpose of securing food. Indeed, it has been proposed that the great expansion of the visual and prefrontal cortices during hominid evolution was driven by the need to develop highly efficient foraging strategies.^156,157^ At a fundamental level, the essence of the superior capabilities of the human brain, including invention, imagination, efficient decision-making, creativity and language, is based on pattern processing.^158^ A prediction of the ‘superior pattern-processing hypothesis’ of human brain evolution is that physical exertion, DER and complex cognitive challenges enhance pattern- processing capability. Direct support for the latter prediction comes from recent studies showing that wheel running enhances spatial pattern separation in mice^159^ and that exercise improves cognitive function across a range of domains in human subjects.^160,161^ ILOD involves progressive deficits in pattern processing resulting from the degeneration of neurons that mediate pattern processing. It is therefore reasonable to conclude that the same challenges that shaped the evolution of the human brain over millions of years can also sustain brain structure and function during aging.

## Future directions and implications for the prevention and treatment of ILOD

While drug companies, neurologists and many researchers who study neurodegenerative disorders emphasize the need to develop and prescribe drugs specific for prototypical ILODs (i.e., AD, FTD, LBD, HSA), the findings reviewed above suggest that the mosaic nature of the molecular and cellular neuropathological landscape of ILODs is more amenable to interventions that stimulate multiple pathways that bolster neuronal plasticity and stress resistance. The advantages of prescriptions for intermittent challenges (exercise, DER and intellectual endeavors) are manifest and include (1) the fact that our brains (and bodies) evolved so as to benefit from the challenges; (2) the relative lack of any adverse side effects; and (3) little or no cost to the patient (Figure 3). The barriers to intermittent challenge-based interventions revolve mostly around the economic and political forces that prevent their implementation, namely the pharmaceutical, processed food and health-care industries. Put simply, the profits of the latter industries would suffer greatly if prescriptions for intermittent challenge-based lifestyles were widely implemented such that far fewer individuals developed chronic diseases including ILOD. Therefore, a major future direction for research on intermittent challenges that engage adaptive stress response pathways is to better understand the specific machinations of the pharmaceutical and food industries that have fostered the rising tide of populations that are encouraged to overeat, and then to take drugs to treat the symptoms of the many ailments they develop.

Challenge–recovery cycles and the term hormesis describe the temporal and quantitative features of intermittent challenges that bolster brain health and resistance to ILOD. The molecular and cellular changes that occur in brain cells during the challenge (exercise, DER and cognitive challenges) and recovery (rest, eating, sleeping) periods are beginning to be understood (Figure 3a). During the challenge, neuronal activity and energy demand increase, and kinases (e.g., CaMKII and AMPK) and transcription factors (e.g., CREB and PGC-1α) are activated. As a consequence, the expression of genes encoding proteins involving autophagy, free radical metabolism and DNA repair is increased. This challenge places the cells in a ‘preservation mode’ in which mammalian target of rapamycin activity and overall protein synthesis are reduced, while pathways that bolster stress resistance are engaged. During the recovery period, protein synthesis increases, mitochondrial biogenesis occurs, and neurite outgrowth, synapse formation and neurogenesis occur. In the absence of challenges (i.e., a ‘couch potato’ lifestyle), the pathways normally activated by the challenges are downregulated, resulting in the accumulation of ‘molecular toxic waste’ including aggregated Aβ, p-Tau, TDP-43 and α-synuclein, and dysfunctional mitochondria. Future research on the effects of challenge–recovery cycles should include (1) expanding and refining an understanding of the molecular and cellular responses of brain cells to different patterns and intensities of challenges, and the impact of individual and combined challenges on functional outcomes in animal models relevant to ILODs; (2) randomized controlled trials to establish the effects of intermittent challenges on brain function and chemistry in healthy subjects, individuals at risk for ILOD, and individuals in the early symptomatic stage of ILOD; and (3) development and implementation of specific prescriptions for intermittent challenge routines to promote and sustain brain health during aging (e.g., Figure 3b).

Hormesis occurs when transient exposure of a cell or organism to a low to moderate level of an agent or condition (e.g., ingestion of a chemical, high temperature, exercise, food deprivation) induces an adaptive/beneficial response, while exposure to higher and/or sustained levels of the agent or condition results in detrimental effects on the cell or organism.^162–164^ Thus, a biphasic dose–response curve is a defining feature of hormesis. In addition to the evidence suggesting that exercise, DER and intellectual challenges may forestall ILOD by hormesis-based mechanisms, it has been shown that chemical challenges can activate adaptive stress response pathways and protect neurons in models relevant to ILOD. Indeed, emerging findings suggest that some chemicals in fruits and vegetables are ‘toxins’ from the perspective of plant evolution—they are noxious phytochemicals that function as natural pesticides/antifeedants. Examples of such neuroprotective ‘hormetic phytochemicals’ include sulforaphane, curcumin, epicatechins and resveratrol.^165^ Considerable further basic and translational research will be required to determine if and to what extent such phytochemicals, or man-made drugs that activate hormetic pathways, can counteract the pathological cascades believed to occur in ILOD. Nevertheless, a broader appreciation of the potential for approaches that engage intrinsic pathways that bolster neuroplasticity and stress resistance may help accelerate the development of viable prophylactic and treatment approaches to halt and reverse the emerging ILOD crisis.
